# The impact of life hardiness, attachment style and personality profile on ptsd vulnerability manifestation in breast cancer patients undergone successful surgical treatment

**DOI:** 10.1192/j.eurpsy.2021.1147

**Published:** 2021-08-13

**Authors:** A. Vasileva, E. Lukoshkina

**Affiliations:** Non-psychotic Disorders Treatment And Psychotherapy, V.M. Bekhterev National Research Medical Center for psychiatry and neurology, saint petersburg, Russian Federation

## Abstract

**Introduction:**

PTSD manifestation is determined by facing extreme life threatening experience going beyond our stress coping skills. The diagnosis of the serious illness like cancer or SARS-2 COVID 19 can be considered as one of the PTSD risk factors. In our clinical practice we have to distinguish the patients groups vulnerable to comorbid PTSD as well as define the psychological factors like good life hardiness, adaptive internal illness image or specific personality profile that can help to cope with disease stress and should be strengthen with psychosocial interventions.

**Objectives:**

After screening with PTSD Trauma Screening Questionnaire and an expert clinical interview aimed to verify the PTSD diagnosis according to ICD-10 criteria 97 breast cancer patients were enrolled in the study, 46 with comorbid PTSD, 51 well coped with stress

**Methods:**

Semi-structured interview, Hardiness Survey questionnaire,Experiences in Close Relationships-Revised (ECR-R) Adult Attachment questionnaire,Impact of Event Scale-R – IES-R, the questionnaire of the internal disease model, Ego-structure test by G. Ammon(ISTA),.

**Results:**

The correlation analysis revealed negative correlation between PTSD diagnosis and hardiness, especially its Involvement, Control, Risk acceptance sub-scales and with the Traumatic event impact score. Deficient-destructive ISTA personality profile had a positive correlation with PTSD and traumatic Impact scores, the strongest correlation were with deficient aggression(r=0,698, p=0.01), destructive anxiety (r=0,674, p=0,01), and deficient internal and external demarcation (r=0,678, p=0,01). The adaptive internal illness image types had a negative correlation with PTSD

**Conclusions:**

Hardiness, maladaptive illness images types and destructive-deficient personality dimension should be the main targets for psychotherapy in comorbid PTSD treatment and prevention

**Keyword:**

life hardiness ptsd breast cancer internal image of the illness construct anxiety social support

